# Metabolic Characterization of Supernatants Produced by *Lactobacillus* spp. With *in vitro* Anti-*Legionella* Activity

**DOI:** 10.3389/fmicb.2019.01403

**Published:** 2019-06-26

**Authors:** Virginia Fuochi, Maria Anna Coniglio, Luca Laghi, Antonio Rescifina, Massimo Caruso, Aldo Stivala, Pio Maria Furneri

**Affiliations:** ^1^Department of Biomedical and Biotechnological Sciences (BIOMETEC), University of Catania, Catania, Italy; ^2^Department of Medical and Surgical Sciences and Advanced Technologies “GF Ingrassia”, University of Catania, Catania, Italy; ^3^Centre of Foodomics, Department of Agro-Food Science and Technology, University of Bologna, Bologna, Italy; ^4^Department of Drug Sciences, University of Catania, Catania, Italy; ^5^Department of Clinical and Experimental Medicine (MEDCLIN), University of Catania, Catania, Italy

**Keywords:** *Legionella pneumophila*, *Lactobacillus* spp., antibacterial activity, metabolomics, metabolome analysis, nuclear magnetic resonance spectroscopy, metabolic pathway analysis

## Abstract

*Legionella pneumophila* is an organism of public health interest for its presence in water supply systems and other humid thermal habitats. In this study, ten cell-free supernatants produced by *Lactobacillus* strains were evaluated for their ability to inhibit *L. pneumophila* strains isolated from hot tap water. Production of antimicrobial substances by *Lactobacillus* strains were assessed by agar well diffusion test on BCYE agar plates pre-inoculated with *L. pneumophila.* Cell-free supernatants (CFS) showed antimicrobial activity against all *Legionella* strains tested: *L. rhamnosus* and *L. salivarius* showed the highest activity. By means of a proton-based nuclear magnetic resonance (^1^H-NMR) spectroscopy, we detected and quantified the *Lactobacillus* metabolites of these CFSs, so to gain information about which metabolic pathway was likely to be connected to the observed inhibition activity. A panel of metabolites with variations in concentration were revealed, but considerable differences among inter-species were not showed as reported in a similar work by Foschi et al. (2018). More than fifty molecules belonging mainly to the groups of amino acids, organic acids, monosaccharides, ketones, and alcohols were identified in the metabolome. Significant differences were recorded comparing the metabolites found in the supernatants of strains grown in MRS with glycerol and the same strains grown in MRS without supplements. Indeed, pathway analysis revealed that glycine, serine and threonine, pyruvate, and sulfur metabolic pathways had a higher impact when strains were grown in MRS medium with a supplement such as glycerol. Among the metabolites identified, many were amino acids, suggesting the possible presence of bacteriocins which could be linked to the anti-*Legionella* activity shown by cell-free supernatants.

## Introduction

Lactic acid bacteria (LAB) are commensal bacteria widely studied for their postbiotic products, a term which indicates the majority of the metabolites referring to soluble factors secreted by bacteria during their cycle-life and/or released after membrane lysis (Aguilar-Toala et al., 2018). Among these by-products, there are both biosurfactants and bacteriocins (Dykes, 1995; Van Hamme et al., 2006). The former are surface-active compounds including glycolipids, lipopeptides, lipoproteins, phospholipids, and fatty acids that reduce surface and interfacial tension in both aqueous solutions and hydrocarbon mixtures (Desai and Banat, 1997). As for bacteriocins, they are cationic and amphipathic small molecules with antagonistic activity usually against microorganisms closely related to the producing strain (Banerjee et al., 2013). For this reason, the bacteriocin-producing microorganisms have developed a specific immunity, mediated by a gene encoding for an immunity protein that protects the producers from their own bacteriocin (Kristiansen et al., 2016). The mechanism of action of bacteriocins produced by Gram positive bacteria is mostly related to the proton motive force (PMF) dissipation and can be further divided into subclasses on the basis of the energy required (Bruno and Montville, 1993).

*Lactobacillus* spp. are well known for their production of antimicrobial compounds, including biosurfactants, bacteriocins and bacteriocin-like peptides (BLIS) (Ruiz et al., 2012; Madhu and Prapulla, 2014; Sharma and Saharan, 2016; Fuochi et al., 2017, 2018).

Nowadays, many of these molecules are partially or entirely characterized (Gautam and Sharma, 2009; Hata et al., 2010; Zhang et al., 2013; Ge et al., 2016), but it has been assumed that every *Lactobacillus* strain is able to produce its own peculiar postbiotics.

In this contest, component medium, *in vitro*, or diet, *in vivo*, have significant impacts on the probiotic function; this is especially notable for individual food components that are present in high quantities and have known relevance to microbial growth and metabolism (Yin et al., 2018). For example, cell growth can be maintained or enhanced upon the addition of glycerol to anaerobic glucose cultures of *Lactobacillus* sp. changing their metabolism (Westbrook et al., 2018).

Moreover, in recent years, postbiotics have attracted significant attention for their potential use for clinical applications (Tsilingiri and Rescigno, 2013), as safe additives for food preservation (Ahmad Rather et al., 2013), and for other applications such as disinfection/emulsification for industrial uses (Meylheuc et al., 2006; Luna et al., 2013). Gram-positive bacteria produce, above all, small cationic peptides weighting less than 6 kDa and narrow range spectrum. Nevertheless, there are exceptions; for instance, some *Lactobacillus* strains have shown activity against pathogens such as *Pseudomonas aeruginosa* and *Klebsiella pneumoniae* (Fuochi, 2016; Fuochi et al., 2017). In the same way, a strain of *Staphylococcus warneri* has shown the production of a little hemolytic peptide (22 amino acids) which inhibited the growth of *Legionella* spp. (Hechard et al., 2005).

*Legionellae* are ubiquitous, and they could live as free-living planktonic forms, as sessile cells within a biofilm (Berlanga and Guerrero, 2016) or as intracellular parasites of protozoans (Hsu et al., 2011). *L. pneumophila*, in particular, is an organism of public health interest for its presence in water supply systems and other humid thermal habitats (e.g., air conditioners, cooling towers, hospital equipment, water fountains, etc.). It is the causative agent of Pontiac fever – a mild febrile illness – and Legionnaire’s disease or legionellosis – an acute and sometimes life-threatening pneumonia, which can occur as a nosocomial or travel-associated infection, sporadically or as part of an outbreak (Diederen, 2008).

At present, US-EPA listed disinfectants for drinking water are all based on the chlorine atom^1^. The efficacy against *Legionella* varies significantly across disinfectants because the bacterium can resist disinfection, especially when inside amoeba or in biofilms (Abdel-Nour et al., 2013).

In the present study, we evaluated ten CFSs produced by *Lactobacillus* strains belonging to different species, capable of producing molecules which had a broad spectrum of inhibition (Fuochi, 2016; Fuochi et al., 2018), for their ability to inhibit *Legionella pneumophila* strains isolated from hot tap water. Furthermore, a preliminary characterization was performed by ^1^H-NMR analysis to identify a panel of molecules whose variations could be associated with the taxonomy (Foschi et al., 2017). It will be useful in correlating lactobacilli with their anti-microbial activity, with the aim of discovering a decontaminant product that would be appropriate for disinfecting water systems.

## Materials and Methods

### Water Sampling and Isolation of *Legionella* Strains

Thirty samples of tap-water were collected throughout a water distribution system without flushing and without flaming, according to the procedures reported in the Italian guidelines for Legionellosis Prevention (Ministero della Salute, 2016). Isolation of *Legionella* was performed in accordance with the standards procedures ISO 11731:1998, as described elsewhere (Coniglio et al., 2018). In brief, 1L of water from each sampling point was filtered using 0.2 μm polycarbonate filter (Sartorius Stedim Biotech GmbH, Goettingen, Germany) and each filter was vortexed for 15 min in 10 mL of the correspondent water sample to detach bacteria. For each concentrated water sample, an aliquot of 5 mL underwent immediate cultural examination while the remaining 5 mL was treated with heat by exposure to 50°C for 30 min. From both concentrated and heat-treated samples, aliquots of 0.1 mL were transferred onto one plate of BCYE agar (Oxoid Ltd., Basingstoke, Hampshire, United Kingdom) and 0.1 mL onto one plate of Glycine Vancomycin Polymyxin Cycloheximide (GVPC) agar (Oxoid). Plates were incubated at 37°C in modified atmosphere (2.5% CO_2_). After 4, 8, and 14 days of incubation, colonies suggestive for *Legionella* grown on BCYE and GVPC were confirmed on the basis of cultural testing (lack of growth on CYE agar, Oxoid) and were subjected to identification using a latex agglutination test with polyvalent antisera (Legionella Latex Test, Oxoid) and monovalent antisera (Biogenetics Srl, Tokyo, Japan). The test allows the separate identification of *L. pneumophila* Serogroup 1 and Serogroups 7-14 and commonly isolated *Legionella* species.

Molecular analysis was performed by real-time PCR (Ministero della Salute, 2016). Briefly, genomic DNA was extracted using Legionella DNA Extraction Kit (4LAB Diagnostics s.r.l., CR, Italy) according to the manufacturer’s instructions. Afterward, DNA amplification and product detection were performed using MIC PCR cycler 1.4 (Bio Molecular System). All reactions were performed using *Legionella pneumophila* PCR Real-Time Kit (4LAB Diagnostics s.r.l., CR, Italy) in a final volume of 20 μL containing 5 μL of GAPDH mix, 0.5 μL of dNTP, 1.5 μL of MgCl, 2.5 μL of buffer, 10.25 μL of water, and 0.25 μL of Taq. The qPCR reaction contained following steps: 2 min at 95°C followed by 30 cycles of 15 sec at 95°C and 45 sec at 95°C. Finally, during amplification, the reporter dye (FAM) was measured. Data were analyzed with micPCR^©^ software 1.4.1. (Bio Molecular System).

The strains of *Legionella pneumophila* were analyzed for their susceptibility to supernatants of *Lactobacillus* strains (Fuochi, 2016; Fuochi et al., 2017; Clinical and Laboratory Standards Institute [CLSI], 2018).

### Production of Antimicrobial Substances by *Lactobacillus* Strains

The antimicrobial substances produced by *Lactobacillus* strains were obtained as previously described: strains of *Lactobacillus* spp., previously isolated as already reported (Fuochi, 2016; Fuochi et al., 2017, 2018), were grown at 33°C in MRS broth (Sigma-Aldrich Co.) without supplements or with 20% glycerol for 72 h. Then, cells were separated by centrifugation (8,000 rpm, 25 min, 4°C) and CFSs were collected after filtration at 0.22 μm. Moreover, CFSs were adjusted at pH 6.8 with NaOH 1M (Toba et al., 1991).

### Evaluation of Antimicrobial Activity

For the evaluation of antimicrobial activity, agar well-diffusion test bioassay was performed with CFSs obtained from the growth in MRS with and without supplement (Fuochi, 2016; Clinical and Laboratory Standards Institute [CLSI], 2018). 100 μL of each CFS were inoculated into wells made on pre-inoculated BCYE agar plates (Buffered Charcoal Yeast Extract Agar, Merck KGaA, Darmstadt, Germany) and inhibitory activity was measured against *Legionella pneumophila* strains (Clinical and Laboratory Standards Institute [CLSI], 2018). The plates were incubated at 37°C for 72 h in a humid environment (2.5% CO_2_) and the inhibition zones were then measured in mm by a caliper.

An internal control was performed using E-tests on BCYE-α (Liofilchem S.R.L., Teramo, Italy) with erythromycin (ERY) and ciprofloxacin (CIP), ranging from 0.016 to 256 μg/mL and 0.002 to 32 μg/mL, respectively. MIC values of ERY and CIP were taken as the lowest concentration of antibiotic at which the zone of inhibition intersected the E-test strip (De Giglio et al., 2015). The experiments were performed six times.

### ^1^H-NMR Analysis

Seven hundred μL of each CFS were added to 100 μL of a D_2_O solution of 3-(trimethylsilyl)-propionic-2,2,3,3-d4 acid sodium salt (TSP, 10 mmol) as internal standard.

This solution was adjusted to pH 7.00 by 1M phosphate buffer solution (PBS). Furthermore, NaN_3_ (2 mM) was added to prevent the proliferation of microorganisms during the analysis.

^1^H-NMR spectra were recorded at 298 K with an AVANCE III spectrometer (Bruker, Milan, Italy) operating at a frequency of 600.13 MHz. Following previous works on a variety of biofluids (Ventrella et al., 2016; Le Guennec et al., 2017; Foschi et al., 2018; Parolin et al., 2018), the signals from broad resonances originating from large macromolecules were suppressed by a CPMG-filter composed of 400 echoes separated by 0.400 ms and created with a 180° pulse of 0.024 ms, for a total filter of 330 ms. The residual water signal was suppressed by presaturation.

As pictorially described in Supplementary Figure S1, the signals were assigned by comparing their chemical shift and multiplicity with Chenomx software data bank (Chenomx Inc., Canada, ver 8.2), with standards (Chenomx Inc., Canada, ver. 10) and HMDB (Human Metabolome Data Base ver. 2) data bank. Spectra were processed for quantification as detailed elsewhere (Foschi et al., 2018). Integration of the signals was performed for each molecule by means of rectangular integration.

### Related Pathway Analysis

Pathway analysis was performed on MetaboAnalyst platform, ver 4.0 (Chong et al., 2018). The most relevant pathways involved were identified by combining the results obtained from the pathway enrichment analysis, as suggested by Wu et al. (2017). Graphs were generated using MetaboAnalyst platform ver 4.0.

### Statistical Analysis

The experiments for antimicrobial activity bioassay were performed six times. Where applicable, data were analyzed by one-way ANOVA with correction for multiple comparisons by Bonferroni.

## Results

### Isolation and Identification of *Legionella* Strains

**FIGURE 1:**
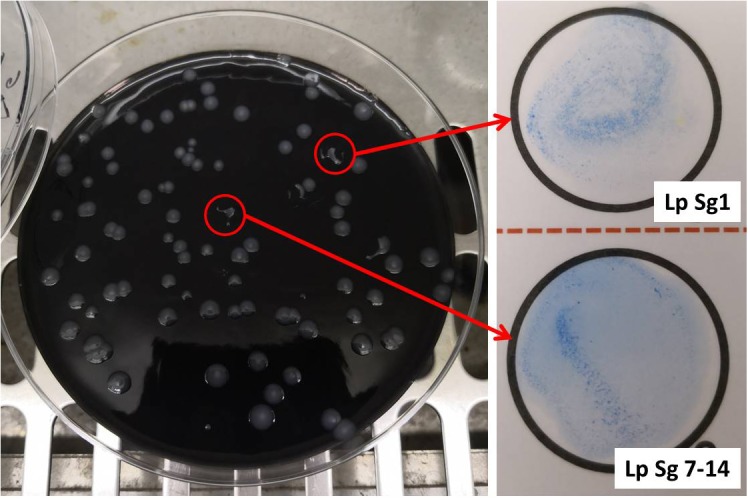
Left, Buffered Charcoal Yeast Extract (BCYE) agar after 14 days of incubation at 37°C in modified atmosphere (2.5% CO_2_). *Legionella* colonies appeared white-gray, shiny, round in shape with whole edges; Right, identification of *Legionella pneumophila* using latex agglutination test with mono (Lp Sg1) and polyvalent antisera (Lp Sg 7–14).

**FIGURE 2:**
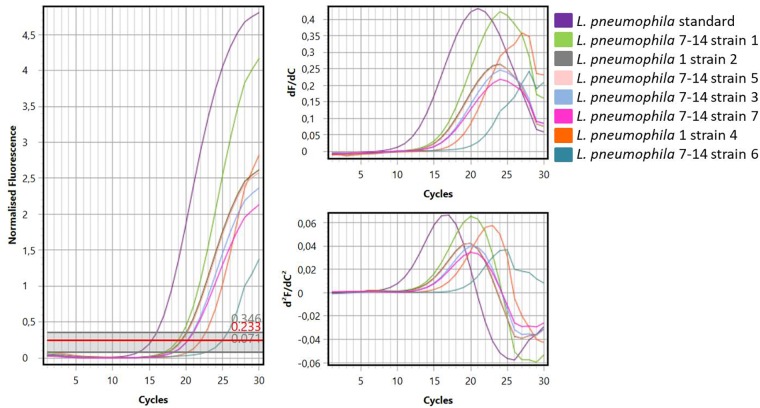
Curves generated by amplification of standard Legionella DNA and DNA samples extracted from *Legionella* strains.

Figure 1 showed one plate of BCYE agar (Oxoid Ltd., Basingstoke, Hampshire, United Kingdom) after 14 days of incubation (on the left) and the subsequent identification of presumptive *Legionella* spp. using latex agglutination test (on the right) with mono and polyvalent antisera (Legionella Latex Test, Oxoid). Agglutination of the blue polystyrene ‘latex’ particles within 1 min was considered a positive reaction. On the 30 hot water samples collected, 7 strains of *L. pneumophila* were detected: namely 5 strains belong to Sg 7–14 and 2 strains belong to Sg 1. Moreover, amplification curves obtained by RT-PCR as a second confirmation of identification were showed in Figure 2.

### Evaluation of Antimicrobial Activity

CFSs of lactobacilli grown in MRS without supplement did not show activity (data not shown). Instead, CFSs obtained from growth with glycerol showed antimicrobial activity against all *Legionella* strains tested. This activity was assessed by inhibition zones on the plates, indicating the lack of growth of *Legionella* in the presence of these supernatants (Table 1). The major inhibition effects were assessed for AD2 *L. rhamnosus* (30 mm) and ML1 *L. salivarius* (30 mm) against 1 Lp 7–14 strain, as well as for AD2 *L. rhamnosus* against 3 Lp 7–14.

**Table 1:** Zones of inhibition measured in mm (diameter) caused by cell-free supernatants of *Lactobacillus* spp. (grown in MRS with the supplement glycerol) against *Legionella* spp.

	Inhibition zones (mm)						
**Strains**	**1 Lp 7–14**	**2 Lp Sg 1**	**3 Lp 7–14**	**4 Lp Sg 1**	**5 Lp 7–14**	**6 Lp 7–14**	**7 Lp 7–14**

VV1 *L. crispatus*	21.9 ± 0.1	17.2 ± 0.2	24.2 ± 0.1	22.9 ± 0.1	20.2 ± 0.1	19.1 ± 0.1	23.0 ± 0.1
RB2 *L. crispatus*	20.9 ± 0.1	17.9 ± 0.1	21.8 ± 0.3	20.8 ± 0.1	19.8 ± 0.1	22.1 ± 0.3	25.1 ± 0.1
ML4 *L. fermentum*	24.2 ± 0.2	15.1 ± 0.1	22.9 ± 0.1	21.4 ± 0.2	18.2 ± 0.1	20.8 ± 0.2	23.2 ± 0.3
SA2 *L. gasseri*	23.8 ± 0.2	14.8 ± 0.2	22.7 ± 0.3	21.2 ± 0.2	17.8 ± 0.2	21.1 ± 0.1	23.2 ± 0.2
SL4 *L. gasseri*	24.9 ± 0.1	17.2 ± 0.1	25.2 ± 0.1	22.7 ± 0.3	19.2 ± 0.1	19.3 ± 0.1	21.8 ± 0.3
SA4 *L. gasseri*	23.9 ± 0.1	15.4 ± 0.2	23.2 ± 0.1	20.6 ± 0.1	18.1 ± 0.3	20.3 ± 0.3	22.9 ± 0.2
SA1 *L. jensenii*	16.2 ± 0.2	18.2 ± 0.1	23.7 ± 0.3	15.4 ± 0.2	12.3 ± 0.1	18.6 ± 0.1	24.1 ± 0.1
AD4 *L. rhamnosus*	18.9 ± 0.1	19.8 ± 0.1	21.2 ± 0.1	18.7 ± 0.2	18.8 ± 0.1	21.7 ± 0.1	21.2 ± 0.3
AD2 *L. rhamnosus*	30.2 ± 0.2	11.7 ± 0.3	30.1 ± 0.1	17.3 ± 0.3	11.8 ± 0.3	22.2 ± 0.2	24.2 ± 0.2
ML1 *L. salivarius*	29.9 ± 0.1	13.2 ± 0.1	23.6 ± 0.3	16.4 ± 0.1	11.1 ± 0.2	12.1 ± 0.1	24 ± 0.1


*The experiments were performed six times. Results have been expressed as mean ± SD.*

The activity of ERY and CIP tested against *L. pneumophila* isolates and one reference strain (*L. pneumophila* NCTC 12821) was reported in Table 2. We decided to use E-test on BCYE-α as internal control because it is a rapid and accurate method for routine susceptibility testing of *Legionella* spp., as previously described (Bruin et al., 2012; De Giglio et al., 2015).

**Table 2:** MIC values of ERY and CIP expressed as μg/mL for *L. pneumophila* isolates for positive internal control.

Drug	1 Lp 7–14	2 Lp Sg 1	3 Lp 7–14	4 Lp Sg 1	5 Lp 7–14	6 Lp 7–14	7 Lp 7–14	Lp sg NCTC 12821
ERY	0.125	0.094	0.125	0.094	0.125	0.125	0.125	0.06
CIP	0.250	0.250	0.125	0.250	0.250	0.250	0.125	0.125


MIC values of ERY were lower than CIP, but there was no significant difference among the MIC values of environmental isolates and NCTC strain.

### ^1^H-NMR Analysis

Thirty-two molecules were identified and quantified in the metabolome of the CFSs produced by ten strains of *Lactobacillus* grown in MRS with glycerol (Supplementary Figure S1). These metabolites belong mainly to families of organic acids, ketones, alcohols, amino acids, and monosaccharides (Table 3). The quantities of the MRS components were reported in Table 4. As done in previous works, the analysis of the metabolic study was carried out on a single sample of CFS, only once (Foschi et al., 2017, 2018; Parolin et al., 2018). Nevertheless, to estimate the measurements reproducibility, the quantification procedure was repeated three times and from a preliminary investigation the median coefficient of variation for the quantified molecules was found to be 3.37.

**Table 3:** Quantification of metabolites detected by ^1^H-NMR of the ten cell-free supernatants produced by *Lactobacillus* spp. grown in MRS with glycerol (20% v/v) expressed as mmoL.^a^

	VV1	RB2	ML4	SA2	SL4	SA4	SA1	AD4	AD2	ML1
Metabolites										
**Organic acids (alcohols, aldehydes, and ketones)**										
1.3-Dihydroxyacetone	0.1160	0.0914	–^b^	0.1733	0.0667	0.1601	0.1243	0.0333	–	–
Acetate	–	–	11.6086	–	–	–	–	–	–	5.4060
Acetoin	–	–	0.0153	0.1002	–	0.0311	–	0.0935	0.4773	0.0572
Acetone	0.0404	0.0374	0.0225	0.0371	0.0337	0.0288	–	0.0468	0.0263	0.0049
Ethanol	1.1489	0.3767	49.1248	0.4433	5.1998	0.5562	12.2024	0.7748	0.4050	10.6746
Formate	–	0.0051	0.4260	–	–	0.0249	–	0.0037	0.0661	0.1656
Lactate	0.1762	0.3521	6.7589	–	0.4303	–	–	0.6278	–	–
Pyruvate	0.0609	0.0298	–	0.0350	–	–	–	0.0014	0.1049	0.0133
**Nucleosides and nucleotides**										
Cytosine	0.0098	0.0080	–	0.0258	0.0103	0.0365	0.0043	–	–	–
Uracil	0.0913	0.1011	0.0697	0.1575	0.1214	0.1535	0.1128	0.1027	0.0624	0.0962
Uridine	0.0051	0.0049	–	–	–	–	–	0.0058	0.0157	–
**Amino acids and derivatives**										
Phenylalanine	–	0.0785	–	0.1060	0.0663	0.0629	0.0432	0.0437	–	–
Tryptophan	–	–	0.0163	0.0209	–	0.0252	0.0391	0.0100	0.0136	0.0373
Tyrosine	0.2713	0.2863	0.2905	0.3510	0.2664	0.3558	0.3111	0.2579	0.3416	0.3533
Alanine	0.3561	0.3899	0.6423	0.8967	0.4111	0.7354	0.7214	0.2853	0.8579	0.8608
Asparagine	0.0414	0.0577	0.2247	0.0852	0.0754	0.0030	0.0880	0.0852	0.1728	0.1414
Aspartate	0.0332	0.0417	0.3168	0.0214	0.0314	0.0836	0.0357	0.0234	0.2074	0.2043
Betaine	0.2029	0.3092	–	0.0917	0.2443	0.0931	–	0.3463	0.0172	–
Isoleucine	0.0613	0.1082	0.1213	0.2106	0.0439	0.2016	0.1212	0.0224	0.1306	0.2062
Methionine	0.0075	0.0136	–	0.0584	–	0.0411	0.0051	–	–	–
Pyroglutamate	0.3944	0.3923	0.5473	0.8159	0.5996	0.7930	0.7598	0.5096	0.8496	0.6239
Sarcosine	0.0453	0.0421	0.0446	0.0578	0.0489	0.0538	0.0171	0.0357	0.0280	0.0392
Threonine	0.2956	0.2910	0.9926	0.4964	0.2264	0.4695	0.4104	0.1056	0.7721	0.8477
Valine	0.2742	0.2156	0.2496	0.5151	0.2613	0.4545	0.3531	0.1043	0.2749	0.4875
**Sugars and derivates**										
Fructose	0.4516	0.4468	–	0.0847	0.5549	0.2224	–	0.5762	0.2541	–
Glucose	7.1884	10.3974	–	3.0722	8.5333	4.0134	1.0097	11.6231	3.3199	–
Maltose	0.1253	0.1195	–	–	0.1396	–	0.1020	0.1604	0.1925	–
Mannitol	–	–	–	–	–	–	–	0.0761	–	–
Ribose	0.0171	0.0192	–	0.0075	0.0151	0.0107	–	0.0247	0.0105	–
Others										
3-Hydroxyphenylacetate	0.0690	0.0485	0.0900	0.0857	0.0757	0.0904	0.0326	0.0575	0.0567	0.1078
Choline	0.0072	0.0115	0.0112	0.0172	0.0117	0.0180	–	0.0117	0.0071	–
2,3-Butanediol	0.0165	0.0042	0.2947	0.0070	0.0241	0.0065	1.2774	0.0060	0.0108	0.2168


*^*a*^Referred to the quantities of substances found in the samples after subtracting the quantities of the components present in the MRS culture medium reported in Table 4; ^*b*^The value of quantification was negative after subtracting the quantities of the components of MRS culture medium.*

**Table 4:** Composition of 69966 MRS Broth (*Lactobacillus* Broth acc. to De Man, Rogosa, and Sharpe) Datasheet Sigma-Aldrich Co.^a^

Ingredients	Grams/Liter
Peptone	10.0
Meat extract	8.0
Yeast extract	4.0
D-(+)-Glucose	20.0
Dipotassium hydrogen phosphate	2.0
Sodium acetate trihydrate	5.0
Triammonium citrate	2.0
Magnesium sulfate heptahydrate	0.2
Manganous sulfate tetrahydrate	0.05

^1^**H-NMR detection**	**mmoL**

1.3-Dihydroxyacetone	0.0752
Acetate	36.7000
Acetoin	0.0248
Acetone	0.0116
Ethanol	0.0386
Formate	0.1120
Lactate	3.0400
Pyruvate	0.0581
Cytosine	0.0294
Uracil	0.1350
Uridine	0.0939
Phenylalanine	1.4000
Tryptophan	0.2440
Tyrosine	0.2281
Alanine	2.3300
Asparagine	0.1240
Aspartate	0.1930
Betaine	0.9820
Isoleucine	0.9150
Methionine	0.3370
Pyroglutamate	0.4340
Sarcosine	0.2500
Threonine	0.4360
Valine	1.5600
Fructose	0.2020
Glucose	8.4600
Maltose	0.1590
Mannitol	6.2500
Ribose	0.0346
3-Hydroxyphenylacetate	0.1020
Choline	0.0786
Propylene Glycol	0.0121


*^*a*^Final pH 6.2 ± 0.2 at 25°C.*

Among the most common molecules found in the literature are lactate, mannitol, glycine, betaine, acetate, ethanol, phenylalanine, formate, uridine, and isoleucine, as well as alanine and valine. Some of these molecules were already present in the culture medium; others are normal metabolites of lactobacilli (e.g., acetate and ethanol) while some are secondary metabolic products.

**FIGURE 3:**
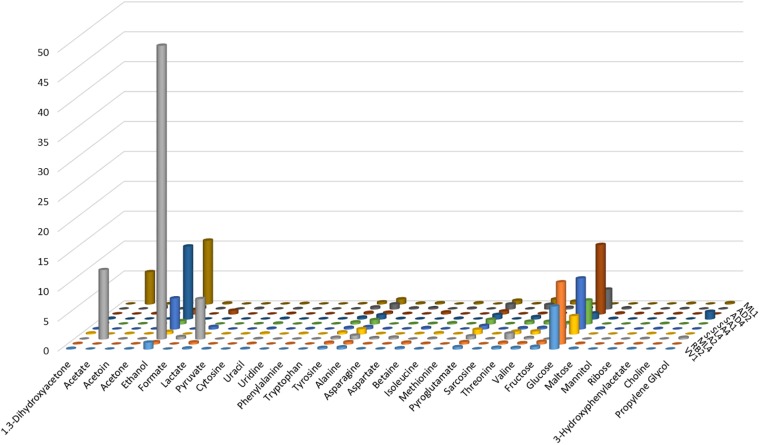
Graphical representation of the metabolite quantities (reported in Table 3) founded in the cell-free supernatants produced by the studied *Lactobacillus* strains grown in MRS with glycerol.

From the examination of the quantities of the identified metabolites, graphically reported in Figure 3, for each of the ten strains, clearly results that ML4 *L. fermentum* strain is particularly rich in ethanol (49.12 mmol), respect to all others strains. Moreover, in ML4 *L. fermentum* strain are present even elevate quantities of acetate (11.60 mmol) and lactate (6.75 mmol), that result absent in the other strains, with the exception of the ML1 *L. salivarius* (acetate = 5.40 mmol); finally, the glucose is completely absent so as in the ML1 *L. salivarius* strain. The quantities of all others metabolites not present particular and relevant differences.

### Related Pathway Analysis

The pathway analysis was performed on MetaboAnalyst platform, to identify the most relevant pathways triggered by the experimental conditions.

**FIGURE 4:**
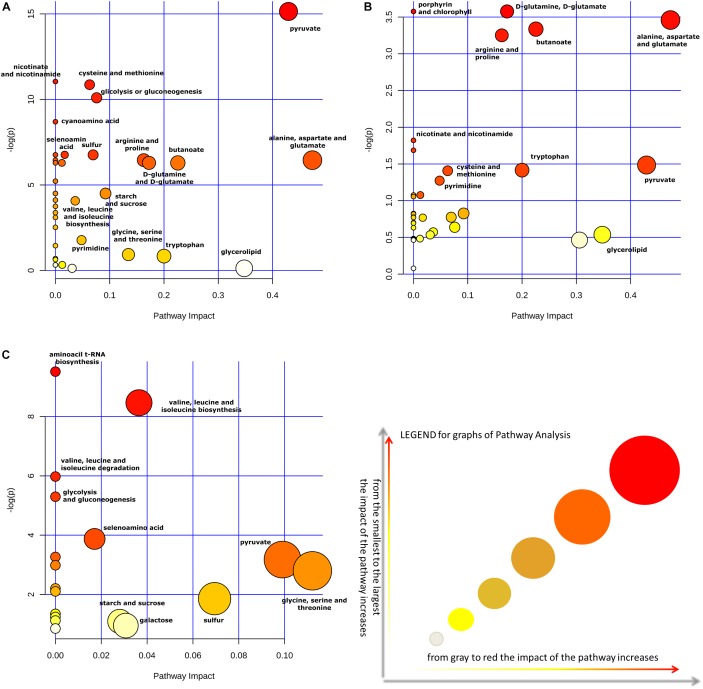
The metabolome view shows all matched pathways, according to the *p*-values, from the pathway enrichment analysis, and pathway impact values from the pathway topology analysis. Impact of pathways on *Lactobacillus* metabolism in presence **(A)** or absence **(B)** of the supplement glycerol in the medium. Panel **(C)** show the impact of pathways considering only the metabolites found in greater quantity in the CFSs. All analyses of metabolite cycles were carried out using Metaboanalyst software (http://www.metaboanalyst.ca). The most significant biochemical pathways were represented with circles in different colors and sizes. In particular, smaller circles or circles in lighter colors (white < gray) indicated that the metabolite is not in the data and it is also excluded from enrichment analysis; larger circles or circle in darker colors (yellow < orange < red) indicated higher-impact metabolic pathways.

Moreover, the impact of glycerol on metabolic pathways of *Lactobacillus* strains was evaluated, by comparing pathway impact values when strains were grown with and without this supplement in the medium. The pathway impact of pyruvate metabolism, D-glutamine and D-glutamate metabolism, porphyrin and chlorophyll metabolism, alanine, aspartate and glutamate metabolism, butanoate metabolism, arginine and proline metabolism were found to be altered when *Lactobacillus* strains were grown in MRS with glycerol (Figure 4A,B). Furthermore, a comparative analysis between all metabolites identified and the most concentrated ones was performed only on CFSs produced with glycerol (Table 5).

**Table 5:** Pathway analysis performed using MetaboAnalyst 4.0 about CFS of *Lactobacillus* strains grown in MRS with glycerol.^a^

Pathway	Match Status	*p*	-log(*p*)	Holm *p*	FDR	Impact
Glycine, serine, and threonine metabolism	2/32	0.0609	2.7981	1.0	0.5889	0.1121
Pyruvate metabolism	2/26	0.0416	3.1774	1.0	0.5182	0.0990
Sulfur metabolism	1/13	0.1557	1.8598	1.0	1.0	0.0694
Valine, leucine, and isoleucine biosynthesis	4/26	0.0002	8.4648	0.0181	0.0091	0.0364
Galactose metabolism	1/37	0.3859	0.9521	1.0	1.0	0.0307
Starch and sucrose metabolism	1/31	0.3345	1.0949	1.0	1.0	0.0280
Selenoamino acid metabolism	2/18	0.0208	3.8727	1.0	0.3619	0.0170
Amino sugar and nucleotide sugar metabolism	1/42	0.4259	0.8535	1.0	1.0	0.0
D-Alanine metabolism	1/3	0.0381	3.2670	1.0	0.5182	0.0
Aminoacyl-tRNA biosynthesis	6/66	7.38E-5	9.5129	0.0064	0.0064	0.0
Phosphonate and phosphinate metabolism	1/4	0.0505	2.9853	1.0	0.5494	0.0
Valine, leucine and isoleucine degradation	3/23	0.0025	5.9758	0.2158	0.0736	0.0
Streptomycin biosynthesis	1/9	0.1103	2.204	1.0	0.9601	0.0
Taurine and hypotaurine metabolism	1/10	0.1219	2.1045	1.0	0.9641	0.0
Glycolysis or Gluconeogenesis	3/29	0.0050	5.2966	0.4207	0.1089	0.0
Phenylalanine metabolism	1/23	0.2599	1.3474	1.0	1.0	0.0
Phenylalanine, tyrosine, and tryptophan biosynthesis	1/23	0.2599	1.3474	1.0	1.0	0.0
Pantothenate and CoA biosynthesis	1/23	0.2599	1.3474	1.0	1.0	0.0
Pentose phosphate pathway	1/26	0.2887	1.2421	1.0	1.0	0.0
Fructose and mannose metabolism	1/30	0.3256	1.1220	1.0	1.0	0.0


*^*a*^The statistical *p*-values were calculated from enrichment analysis; the Holm *p* is the *p*-value adjusted by the Holm-Bonferroni method; the FDR *p* is the *p*-value adjusted using the false discovery rate; the impact is the pathway impact value calculated from pathway topology analysis.*

Finally, Figure 4C allowed to evaluate in greater detail the metabolites found in higher quantities in the CFSs of the strains grown with glycerol thanks to a “zoom” of the biosynthetic pathways used by bacteria in this context. Indeed, as can be seen, the larger and darker circles showed that the biosynthetic pathways of valine, leucine, and isoleucine, as well as the pyruvate, glycine, serine, and threonine pathways had a more significant influence on the metabolism of these lactobacilli.

## Discussion

Nowadays, the prevention of *Legionella* dissemination has an important role in the control of its proliferation in water systems and many chemical disinfection methods have been proposed (Bonetta et al., 2018; Coniglio et al., 2018).

Nevertheless, the research of LAB producers could lead to the discovery of molecules with strong antimicrobial activity for alternative less polluting decontamination method.

In this study, ten CFSs were produced by different species of *Lactobacillus*: *L. crispatus, L. gasseri, L. jensenii, L. fermentum, L. rhamnosus*, and *L. salivarius* grown up with two different culture media. CFSs obtained from the growth in MRS without supplement did not show antibacterial activity; on the contrary, the ones obtained from the growth in MRS with glycerol showed anti-*Legionella* activity emphasizing the important role of nutrition on bacterial metabolism and the consequent production of postbiotics. Furthermore, CFSs produced by AD2 *L. rhamnosus* and ML1 *L. salivarius* followed by SL4 *L. gasseri* showed the strongest activity against two strains of *Legionella pneumophila.* It is interesting to note that the lactobacilli species found to be active against *Legionella* were already demonstrated to have considerable amensalistic characteristics such as resisting bile salts and acidic pH (Fuochi et al., 2015), as well as having the ability to produce biofilms, antibacterial, and antifungal activity (Fuochi et al., 2017, 2018).

In order to understand the subjects involved in the anti*-Legionella* activity ^1^H-NMR analysis was performed. Indeed, ^1^H-NMR spectroscopy proved to be a fast and reliable analytical technique to detect and quantify low molecular weight metabolites in different matrices and biological fluids (Laghi et al., 2014; Parolin et al., 2018), included intracellular and extracellular metabolic profiles of different Gram-positive (e.g., lactobacilli and *Staphylococcus aureus*) and Gram-negative bacteria (e.g., *Acinetobacter baumannii, Pseudomonas aeruginosa*) (Ammons et al., 2014; Chen et al., 2017).

A panel of metabolites with variations in concentration were revealed, but great differences among inter-species were not showed as reported in a similar work (Foschi et al., 2017).

Essential differences were recorded comparing the metabolites found in the supernatants of strains grown in MRS with glycerol and the same strains grown in MRS without this supplement.

Indeed, the presence or absence of glycerol in the medium has transformed the impact of pathways in the metabolism of *Lactobacillus* strains as expected. Acting by this way, in the two cases the role of pyruvate metabolism and alanine, aspartate and glutamate metabolism were changed. In particular, the impact of pyruvate is increased of 10 times; meanwhile, that one of alanine, aspartate and, glutamate is increased twice (Figure 4A,B).

Moreover, metabonomic research considering only the metabolites found in high quantity in the CFSs of *Lactabacillus* grown in MRS with glycerol revealed that glycine, serine, and threonine, pyruvate and sulfur metabolic pathways had a higher impact compared to the same strains grown in MRS without supplement (Figure 4C).

*L. crispatus, L. gasseri*, and *L. salivarius* strains are obligately homofermentative *Lactobacillus* spp. which means they degrade hexoses to pyruvate, which in turn is reduced by lactic fermentation to lactate by the Embden-Meyerhof-Parnas pathway (Willey et al., 2010). Instead, *L. jensenii, L. rhamnosus*, and *L. fermentum* strains are facultatively heterofermentative and obligately heterofermentative *Lactobacillus* spp., respectively. As it is well known, facultatively heterofermentative strains could use both Embden-Meyerhof-Parnas pathway and Warburg-Dickens pathway, meanwhile obligately heterofermentative strains only the Warburg–Dickens pathway (Nelson et al., 2017). The result is the production of pyruvate and lactate as well as ethanol and acetate molecules, consistently with the high concentrations detected by ^1^H-NMR. Whereas the antibacterial activity of these molecules is renowned for a long time (Houtsma et al., 1993; Oh and Marshall, 1993; Lee et al., 2002; Wang et al., 2015; Stanojevic-Nikolic et al., 2016), our attention was focused on other molecules identified by metabolomics analysis such as uridine, phenylalanine, tyrosine, and betaine.

For instance, Yamaguchi et al. (2015) have synthesized uridine derivatives containing isoxazolidine which have shown good activity against *H. influenzae* ATCC 10211 and moderate activity against *E. faecalis* SR7914 with resistance to vancomycin. Regards both tyrosine and phenylalanine, analogous cationic surfactants were synthesized, as ester derivatives, and analyzed; their antibacterial action was more pronounced on Gram-positive strains compared to Gram-negative ones (Joondan et al., 2014). Furthermore, benzyl derivatives of betaine showed bacteriostatic and bactericidal activity against both Gram-positive and Gram-negative strains (Cosquer et al., 2004).

Based on these results, many amino acids as well as other metabolites with recognized antibacterial activity were present in our CFSs. Besides, these molecules were only the small “free ones” detected by ^1^H-NMR, due to the applied CPMG-filter.

The presence of many amino acids could suggests a possible participation of a peptide or a protein in the antibacterial activity, hypothesis already partially confirmed by a previous work (Fuochi et al., 2018). In fact, in that study, the antimicrobial activity of these CFSs were inactivated by enzymes such as trypsin and proteinase K suggesting the presence of proteinaceous molecules, and for this reason we referred to them as BLIS. Nevertheless, it is important to remember, as previously said, that other compounds can be potentially toxic to specific microbes (Singh et al., 2007). Indeed, several studies reported the potential of lactobacilli as microbial surfactants which play an important role as no-conventional antibacterial products against various pathogens (Velraeds et al., 1996; Tahmourespour et al., 2011; Cornea et al., 2016; Sharma and Saharan, 2016).

In the category of biosurfactants are included low molecular-weight compounds such as lipopeptides and glycolipids (Sharma and Saharan, 2016). Anti-*Legionella* activity has already been demonstrated by similar bacterial products, for instance a strain of *Bacillus subtilis* produces the surfactin, a lipopeptide that successfully eliminated 90% of a 6-day-old biofilm (Loiseau et al., 2015). Moreover, Héchard et al. (Hechard et al., 2005), described a new bacteriocin secreted by *S. warneri* RK with anti-*Legionella* activity. Then, sugars, amino acids, and organic acids reported in Table 3 could be part of this kind of molecules.

Although, the full characterization of the molecules present in CFSs has not yet occurred, this is one of the few papers describing an active microbial postbiotic against *L. pneumophila*. Certainly, our results represent the starting point for more in-depth studies, and analysis by MALDI MS are in progress in order to thoroughly investigate and characterize the postbiotic molecule involved in the antimicrobial activity of *Lactobacillus* CFSs with the aim of studying the appropriate method for the production on large-scale of this new eco-disinfectant for the water systems.

## Author Contributions

VF and PF conceived the study. VF, LL, MAC, AR, and PF provided the methodology. VF, LL, and AR developed the software. VF, MAC, LL, and MC curated the data. VF, MC, and AS wrote the original draft of the manuscript. VF, MC, AS, and PF reviewed, wrote, and edited the manuscript.

## Conflict of Interest Statement

The authors declare that the research was conducted in the absence of any commercial or financial relationships that could be construed as a potential conflict of interest.

## Footnotes

1 http://water.epa.gov/drink/contaminants/basicinformation/disinfectants.cfm

## Abbreviations

BCYE: Buffered Charcoal Yeast Extract
BLIS: bacteriocin-like inhibitory substances
CFS: cell-free supernatant
MIC: minimal inhibitory concentration
MRS: De Man, Rogosa, and Sharpe
NMR: nuclear magnetic resonance
