# Supplementary material for: Metabolic Characterization of Supernatants Produced by Lactobacillus spp. With in vitro Anti-Legionella Activity
Source: Front Microbiol. 2019 Jun 26;10:1403. doi: 10.3389/fmicb.2019.01403 (PMC6606692; doi:10.3389/fmicb.2019.01403)
Supplement: Supplementary file 1 [file Table_1.DOCX]

Supplemental Material

**Metabolic characterization of supernatants produced by *Lactobacillus* spp with *in vitro* anti-*Legionella* activity**





FIGURE S1. Pictorial representation of the assignment of the signals from 1H-NMR spectra. Five spectra from strains grown without (black) and five from strains grown with (red) glycerol are portrayed. The name of the molecules quantified in table 3 is reported above (or near) the signal employed for quantification.
